# Short prokaryotic Argonaute system repurposed as a nucleic acid detection tool

**DOI:** 10.1002/ctm2.1059

**Published:** 2022-09-26

**Authors:** Ana Potocnik, Daan C. Swarts

**Affiliations:** ^1^ Laboratory of Biochemistry Wageningen University Wageningen The Netherlands

1

Detection of the presence of specific nucleic acid sequences is an important molecular diagnostics method: It facilitates the identification of pathogens in samples ranging from agriculture and food to medicine,^1^ and can be used for genotyping purposes in order to identify single nucleotide polymorphisms (SNPs) or other mutations related to the development of disease.^2^ Since its invention in 1985, polymerase chain reaction (PCR)^3^ has become a widely used nucleic‐acid‐based molecular diagnostic tool. PCR‐based detection methods generally rely on sequence‐specific amplification of a target sequence, during which the presence of the target sequence can be determined by following the generation of a fluorescent signal.^1^ Its robustness, specificity, and sensitivity facilitated its development to become the gold standard for the detection of nucleic acids. However, PCR requires trained personnel and specialized laboratory equipment such as thermocyclers and is therefore unsuitable for nucleic acid detection outside of laboratory settings. Accessible, rapid, and sensitive tools that can be used on the field, at the point‐of‐care, or at home, will greatly benefit diagnostic purposes. The need for thermocyclers required for PCR has been circumvented by the development of isothermal amplification techniques, during which the target sequence is amplified at a constant temperature.^4^ Akin to PCR, also this technique can be combined with fluorescence and colourimetric detection methods.^4^ However, many isothermal amplification techniques face challenges including high background signal, complex primer design, and false‐positives^4^ which slowed down their broader commercialization.

The need for rapid, robust in‐field solutions was underlined during the severe acute respiratory syndrome coronavirus 2 pandemic, which resulted in the accelerated development and application of novel programmable nucleic‐acid detection tools based on repurposed prokaryotic immune systems.^5^ Certain prokaryotic immune systems, including CRISPR‐Cas systems, have evolved to use small RNA guides to specifically bind complementary viral DNA or RNA target sequences. This generally triggers nuclease activity that degrades the viral DNA or RNA. These systems can be isolated and reprogrammed with synthetic guide RNAs with sequences of choice to target specific nucleic acid sequences in samples of interest.^5^ Upon target binding, the triggered nuclease activity can be exploited to convert specific substrates to easy‐to‐detect (fluorescent) products. Although these systems are highly selective, their sensitivity is usually below clinically relevant levels. However, isothermal amplification can be used to increase the target sequence concentration, while relying on the CRISPR‐based tool for selectivity. Combined, these techniques provide a sensitive and selective sequence detection method that could be used outside laboratory settings^6^

Recent studies have uncovered a large number of previously unknown prokaryotic immune systems. Amongst these are prokaryotic Argonaute proteins (pAgos), which also can be programmed with RNA (or DNA) guides to bind complementary nucleic acids. pAgos that cleave their targets upon target binding (“slicers”) have been repurposed to deplete specific abundant sequences, enhancing the sensitivity of PCR‐mediated nucleic acid detection.^7^ Like CRISPR‐Cas systems, pAgos are highly diversified and associated with a wide variety of auxiliary proteins.^8^ Understanding the molecular mechanisms of these diversified systems provides opportunities for the development of novel molecular diagnostics tools.

In a recently published study, we shed light on the function and mechanisms of a novel pAgo‐based prokaryotic immune system: Short prokaryotic Argonaute/TIR‐APAZ (SPARTA; Figure 1).^8^ SPARTA systems are comprised of a catalytically inactive short pAgo and a TIR‐APAZ protein that form a heterodimeric complex. In the complex, short pAgo acts as the ‘sensor’ that uses guide RNAs to bind single stranded (ss)DNA targets. Upon target binding, NAD(P)ase activity of the TIR‐APAZ ‘effector’ is unleashed. In vivo, SPARTA targets invading plasmid DNA, triggering cellular depletion of the metabolites NAD^+^ and NADP^+^, resulting in cell death. In this way plasmid‐invaded cells are removed from the bacterial population, providing population‐level immunity to invading DNA (Figure 1).

**FIGURE 1:**
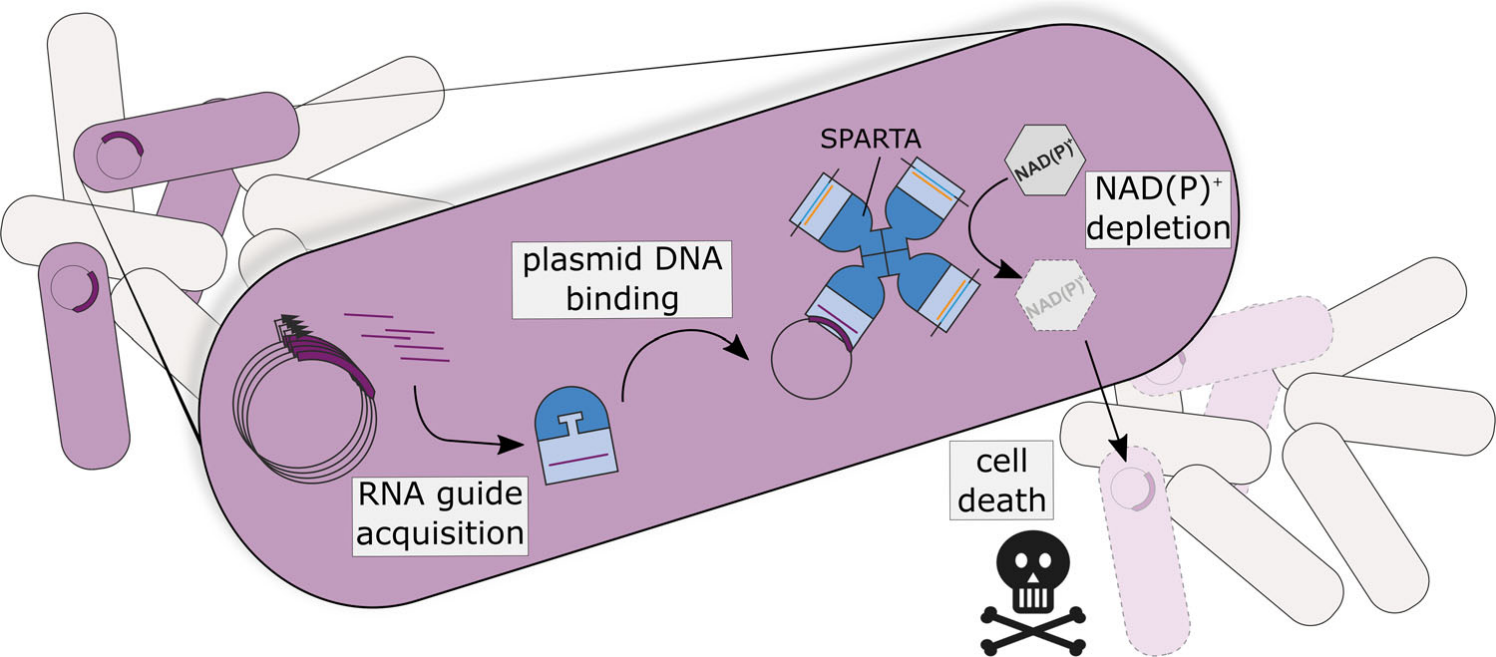
Short prokaryotic Argonaute/TIR‐APAZ (SPARTA) is a bacterial immune system that provides its bacterial host with population‐level immunity against invading plasmids. SPARTA acquires guide RNAs from transcripts of plasmid‐encoded genes to bind complementary plasmid DNA targets. Upon target DNA binding, SPARTA oligomerizes and becomes catalytically activated, after which its NAD(P)ase is unleashed, depleting the cell of NAD(P)^+^. This triggers cell death in plasmid‐invaded cells, removing plasmid‐invaded cells from the bacterial culture

The molecular mechanism of SPARTA offers a unique possibility to repurpose it as a nucleic acid detection tool (Figure 2).^8^ To this end, SPARTA is isolated and reprogrammed with 15–25 nucleotide long synthetic RNA guides, and incubated with an analogue of NAD^+^: etheno‐NAD (ɛ‐NAD). Upon detection of ssDNA targets by SPARTA ɛ‐NAD is converted into easy‐to‐detect fluorescent ɛ‐ADPR. We show that without pre‐amplification step SPARTA has a nanomolar‐level sensitivity to bind targets, comparable to CRISPR‐based tools.^6^ To facilitate detection of double stranded DNA at clinically relevant attomolar levels, we combined SPARTA‐based detection with a modified PCR strategy previously exploited for a CRISPR‐based tool (Figure 2).^9^ To this end, the target DNA is PCR amplified with primers of which one contains a 5′‐end phosphorothioate modification. Upon incubation of the PCR product with a T7 exonuclease, the non‐modified strand is degraded, while the phosphorothioate‐modified strand is protected and acts as a ssDNA target that is detectable by SPARTA.

**FIGURE 2:**
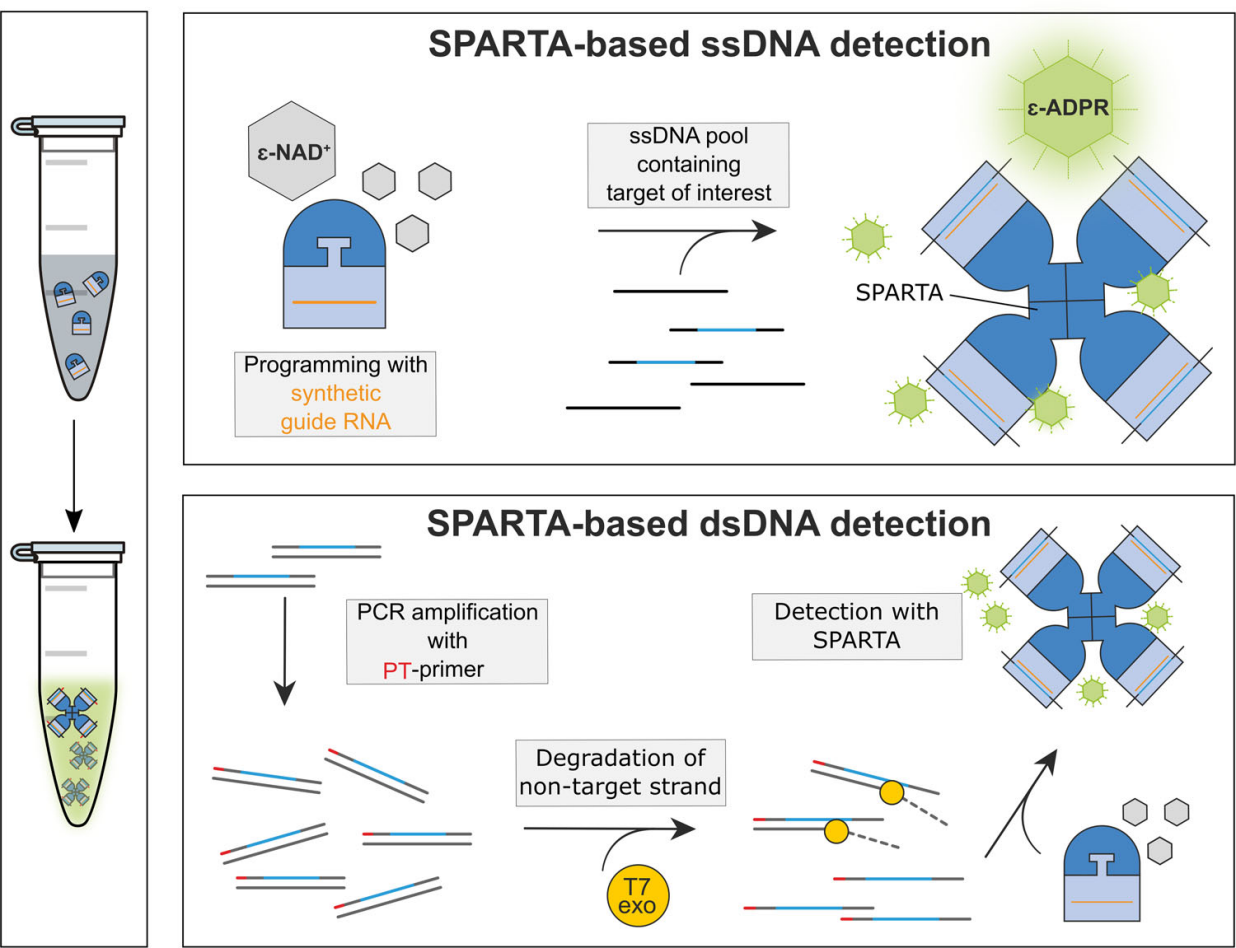
Schematic representation of the Short prokaryotic Argonaute/TIR‐APAZ (SPARTA)‐based nucleic acid detection tool. Left panel: SPARTA can be isolated and re‐programmed with guide RNAs of choice to detect DNA in vitro. Top panel: SPARTA‐based ssDNA detection. SPARTA can be provided with a synthetic RNA guide with a sequence of choice. This allows the detection of complementary single‐stranded DNA target sequences in pools of DNA. The presence of specific DNA sequences can be detected by supplementing the reaction with etheno‐NAD (ɛ‐NAD), an analogue of NAD^+^ that is converted into fluorescent ɛ‐ADPR by activated SPARTA complexes. Bottom panel: SPARTA‐based dsDNA detection. To enable dsDNA detection and to increase the sensitivity of SPARTA‐based detection, target DNA can be specifically amplified by PCR using a phosphorothioate (PT) forward primer and an unmodified reverse primer. Upon incubation with T7 exonuclease, the unmodified strand is degraded, leaving ssDNA fragments containing the target sequence that can be detected by SPARTA

To determine its specificity, SPARTA was incubated with targets that have single‐ and double‐mismatches with the guide RNA.^8^ SPARTA is insensitive to single mismatches but exhibits sensitivity for double mismatches; introducing two consecutive mismatches in the middle region of the guide drastically lowers or completely abolishes SPARTA activity. The insensitivity for single mismatches could be exploited for SPARTA‐mediated detection of pathogens with high mutation rates while maintaining specificity. Furthermore, its sensitivity for double mismatches provides a possibility to re‐program SPARTA for single nucleotide polymorphism genotyping: by introducing a mismatch into the guide on a position immediately followed by the expected SNP, SPARTA will detect mutated sequences, but would not be activated in the presence of wild‐type targets.

Combined, we provide proof of principle that SPARTA can be repurposed for programmable sequence‐specific detection of nucleic acids. We envision that, akin to CRISPR‐based tools,^6^ SPARTA can be combined with isothermal amplification techniques and that the fluorescent signal generated in the presence of target sequences could be detected by hand‐held devices.^10^ This would facilitate the development of sequence‐specific SPARTA‐based molecular diagnostic tools that can be used outside of laboratory settings. The discovery and repurposing of SPARTA once again show that prokaryotic immune systems are a treasure trove of new biotechnological advances. Fundamental characterization of their functional mechanisms paves the way for a new generation of molecular tools.

## CONFLICT OF INTEREST

Ana Potocnik and Daan C. Swarts (together with Balwina Koopal) submitted a patent application regarding the utilization of short pAgo systems for nucleic acid detection.
